# Qualitative and quantitative analysis of solid renal tumors by high-frame-rate contrast-enhanced ultrasound

**DOI:** 10.1186/s40644-024-00788-3

**Published:** 2024-10-15

**Authors:** Hailan Wu, Jiayu Shi, Long Gao, Jingling Wang, WenXin Yuan, WeiPing Zhang, Zhixing Liu, Yi Mao

**Affiliations:** 1 Department of Ultrasound, The First Affiliated Hospital, Jiangxi Medical College, Nanchang University, Nanchang, China; 2 The First Clinical Medical College, Jiangxi Medical College, Nanchang University, Nanchang, China; 3 School of Advanced Manufacturing, Nanchang University, Nanchang, China

**Keywords:** Contrast-enhanced ultrasound, High-frame-rate, Qualitative and quantitative, Renal tumors

## Abstract

**Objective:**

To analyze the characteristics of high-frame-rate contrast-enhanced ultrasound (H-CEUS) in solid renal tumors using qualitative and quantitative methods.

**Methods:**

Seventy-five patients who underwent preoperative conventional ultrasound (US), conventional contrast-enhanced ultrasound (C-CEUS), and H-CEUS examination of renal tumors were retrospectively analyzed, with a total of 89 renal masses. The masses were divided into the benign (30 masses) and malignant groups (59 masses) based on the results of enhanced computer tomography and pathology. The location, diameter, shape, border, calcification, and color doppler blood flow imaging (CDFI) of the lesions were observed by US, and the characteristics of the C-CEUS and H-CEUS images were qualitatively and quantitatively analyzed. The χ² test or Fisher’s exact probability method was used to compare the US image characteristics between the benign and malignant groups, and the image characteristics of C-CEUS and H-CEUS between the benign and malignant groups. Moreover, the nonparametric Mann-Whitney test was used to compare the differences in C-CEUS and H-CEUS time-intensity curve (TIC) parameters.

**Results:**

Significant differences in gender, surgical approach, echogenicity, and CDFI were observed between the malignant and benign groups (*p =* 0.003, < 0.001, < 0.001, = 0003). Qualitative analysis also revealed significant differences in the mode of wash-out and fill-in direction between C-CEUS and H-CEUS in the malignant group (*p* = 0.041, 0.002). In addition, the homogeneity of enhancement showed significant differences between the two contrast models in the benign group (*p* = 0.009). Quantitative analysis indicated that the TIC parameters peak intensity (PI), deceleration time (DT) /2, area under the curve (AUC), and mean transition time (MTT) were significantly lower in the H-CEUS model compared to the C-CEUS model in both the benign and malignant groups. (all *p* < 0.001). In contrast, ascending slope of rise curve (AS) was significantly higher in the H-CEUS model compared to the C-CEUS model in the malignant group (*p* = 0.048).

**Conclusions:**

In renal tumors, H-CEUS shows clearer internal enhancement of the mass and the changes in the wash-out period. The quantitative TIC parameters PI, DT/2, AUC, and MTT were lower in H-CEUS compared to C-CEUS. Both the quantitative and qualitative analyses indicated that H-CEUS better displays the characteristics of solid renal masses compared with C-CEUS.

## Introduction

Renal tumor incidence is rising, with as much as 80–90% of cases of renal cell carcinoma arising from the tubular epithelium of the renal parenchyma [1]. Most of these patients eventually require partial or total nephrectomy. In contrast, renal angiomyolipoma (AML), which is also a solid mass, is the most prevalent benign kidney tumor [2]. However, these patients rarely need to undergo nephrectomy, requiring only resection of the tumor or ablation. The primary goal of imaging renal masses is to differentiate renal cell carcinoma from benign conditions, which can be challenging in some cases. Research has reported that about 20% of renal tumors removed during surgery are benign [3], which increases healthcare expenses and raises the risk of surgery. Moreover, early-stage renal cell carcinoma and AML present nonspecific clinical characteristics [4], highlighting the importance of preoperative imaging in determining the surgical approach.

Contrast-enhanced computer tomography (CECT) has been recommended by many American societies as the investigation of choice for the assessment of patients with non-narcolemmal hematuria [5, 6], but it has the disadvantages of radiation, contrast allergy, and nephrotoxicity of contrast agents. Contrast-enhanced magnetic resonance imaging (CEMR) offers better soft-tissue visualization compared to CECT [7] and is radiation-free, and is recommended by the European Association of Urology (EAU) [8], However, CEMR is expensive, prone to motion artifacts, and unsuitable for patients with claustrophobia and allergy to gadolinium contrast agents.

Ultrasound is a simple, radiation-free examination, and is the initial screening tool of choice for the evaluation of renal lesions; yet, conventional ultrasound has a low sensitivity. Contrast-enhanced ultrasound (CEUS) refers to a combination of conventional ultrasound and contrast agent, which displays the perfusion difference between the lesion and the organ through the nonlinear harmonic signal produced by the contrast agent [9]. In addition, the contrast agent is not metabolized by the kidneys but is exhaled as a gas through the lungs, which allows its use in patients with renal insufficiency; moreover, the incidence of allergy to CEUS contrast agent is very low. Some studies have shown that CEUS may be as accurate or even more accurate than CECT or CEMR in the diagnosis of renal masses [10]. However, masses exhibiting similar enhancement characteristics, such as rapid enhancement or rapid wash-out on CEUS, represent significant challenges in differentiating between benign or malignant masses. Compared to traditional contrast-enhanced ultrasound (C-CEUS), which has a frame rate of 10–15 Hz, high-frame-frequency ultrasonography (H-CEUS) has a frame rate of 50–80 Hz [11]. This effectively improves the temporal resolution of the image and enhances its ability to depict the blood flow characteristics of the mass. Therefore, the time-intensity curve (TIC) can more intuitively and quantitatively show the perfusion characteristics of the mass [12, 13].

This study intended to explore the imaging features of solid renal tumors on H-CEUS by retrospectively comparing them to C-CEUS image features from 89 solid renal masses. The imaging features were combined with a quantitative analysis of TIC parameters.

## Methods

### Objects of study

Seventy-five patients who underwent CEUS examination of renal masses at the First Affiliated Hospital of Nanchang University from December 2022 to June 2024 were selected, with a total of 89 renal masses. The inclusion criteria were (1) age ≥ 18 years; (2) two-dimensional gray-scale ultrasound showing a solid renal mass; (3) a lesion diameter greater than 1 cm; (4) examined by C-CEUS and H-CEUS and could be quantitatively analyzed; (5) a degree of fit of the TIC curve (GOF) > 0.7. The exclusion criteria were as follows: (1) patients suffering from other renal diseases; (2) patients whose respiratory range was too large resulted in poor image storage and could not be analyzed; (3) patients with deep masses in whom inadequate ultrasound penetration prevented the interpretation of the CEUS images. Crude needle aspiration or postoperative pathology were used to confirm the diagnosis for malignant masses; enhanced CT, postoperative pathology, or crude needle aspiration and 3–6 months of follow-up were used to confirm the diagnosis for benign masses. Based on the enhanced CT and pathology results, the masses were categorized into the benign (30 masses) and malignant (59 masses) groups. Our Medical Ethics Committee (IIT2023174) approved the trial, and each participant gave their informed consent.

### Inspection techniques

A Mindray Resona R9 diagnostic ultrasound machine was used, with a SC 5-1U probe and a frame rate of 3–5 MHz. CEUS was performed using an ultra-broadband nonlinear imaging technique. The frame rate was 10 Hz for C-CEUS imaging and 50–65 Hz for H-CEUS. In addition, the mechanical indices used for both imaging methods were 0.06–0.08. SonoVue (Bracco, Italy) was the ultrasonography contrast agent utilized. Prior to the examination, a suspension was created by adding 59 mg of SonoVue to 5 mL of 0.9% sodium chloride solution, shaking thoroughly, and mixing.

### Methods of operation

Depending on the location of the mass, the lateral or prone position was adopted to clearly show the long-axis portion of the mass and the surrounding normal tissues of the kidney. The C-CEUS examination was performed first, with 1 ml of SonoVue suspension being injected through the superficial vein of the elbow, followed by 5 ml of 0.9% sodium chloride solution into the tube. An interval of more than ten minutes was allowed to ensure the complete disappearance of the contrast agent, and the The H-CEUS examination was carried out. All the dynamic images were continually captured and saved for 3 min to 5 min. Two physicians with over five years of CEUS experience examined all ultrasound picture. The pathology and enhanced CT results were hidden from the doctors.

### Conventional ultrasound image characterization

The conventional ultrasound (US) yielded the following features. (1) nodal laterality; (2) nodal location; (3) nodal echogenicity; (4) nodal boundary; (5) nodal shape; (6) Color Doppler blood flow imaging (CDFI) grading, which was classified as grade 0 (no blood flow seen in the tumor), Grade I (small amount of 1 ∼ 2 stellate blood flow), Grade II (moderate blood flow in the form of 3–4 stellate or short fascicles), and Grade III (abundant blood flow in the form of 2–3 or more colored blood flow in the form of reticulation or branching); (7) calcification (present or absent); (8) tumor diameter.

### Qualitative analysis of CEUS images

The following CEUS characteristics were noted. (1) wash-in mode: according to the time of appearance of contrast in the lesion area compared to the surrounding normal renal cortex, it was divided into fast wash-in, iso-wash-in and slow wash-in; (2) wash-out mode: according to the focal intracellular contrast clearance time in the lesion area compared to the surrounding normal renal cortex, it was divided into fast wash-out, iso-wash-out and slow wash-out ; (3) peak intensity: according to the intensity of enhancement in the lesion area compared to the surrounding normal renal parenchyma, it was divided into high enhancement, iso-enhancement and low-enhancement; (4) homogeneity of enhancement (differentiating between homogeneous and heterogeneous enhancement based on the distribution of the intensity of the enhancement); (5) no enhancement (no visible contrast enhancement in the lesion); (6) fill-in direction: according to whether the focal enhancement first appears in the center or the peripheral parts, it is divided into centripetal enhancement, centrifugal enhancement, and entirety enhancement. (7) boundary after enhancement (the degree to which it was easy to distinguish the lesion from the surrounding normal renal cortex); (8) enhancement range: according to the enhanced area produced by the contrast agent at the lesion site and its surrounding tissues, it is divided into an enlarged area and an unenlarged portions; (9) pseudocapsule (whether circumferential hyperenhancement around the nodule was observed).

### Quantitative analysis of CEUS images

The TIC curve was quantified using the analysis software from the Myers Resona R9 color Doppler ultrasound diagnostic instrument, and the region of interest (ROI) was placed within the renal lesion. The ROI was placed by carefully avoiding large vessels and necrotic areas; in inhomogeneously enhanced lesions, the region with the highest enhancement intensity was used (avoiding circumferential enhancement areas). The main output parameters included the following indexes: (1) Arrival time (AT), referring to the time point when the contrast agent started to appear; (2) Peak intensity (PI), referring to the highest intensity of contrast agent perfusion, with the highest intensity of the surrounding tissues as 100%; (3) Time to peak (TTP), referring to the time when the contrast agent starts to perfuse and reaches the PI; (4) Ascending slope of rise curve (AS), referring to the slope of the curve between the onset of perfusion and the peak of the lesion; (5) Deceleration time/2 (DT/2), the time required for the peak intensity to be reduced to half; (6) Maximum slope of decline curve (DS), the slope between the two spots on the curve where the lesion fades and disappears. (7) Area under the curve (AUC), the area under the time-intensity curve of the contrast process; (8) Mean transition time (MTT), the time between the point of arrival of the contrast agent and the point of contouring of the contrast agent. (9) GOF indicates the degree of fit between the fitted curve and the original curve, with a range of 0 to 1, with 1 indicating a perfect fit between the fitted curve and the original curve.

### Statistical methods

SPSS 26.0 (IBM Corporation, Armonk, NY, USA) statistical software was used to process the above data. The variables age and tumor diameter conformed to a normal distribution and the differences between the benign and malignant groups were compared using the *t*-test. The number of cases was expressed for gender, nodal features, and gray-scale ultrasonography characteristics that were the count data. The differences between the benign and malignant groups were compared using the *χ*^*2*^ test or Fisher’s exact probability method. In addition, The two CEUS modalities’ enhancement characteristics were count data, which could also be stated as the number of cases. The enhancement characteristics of the benign and malignant groups’ H-CEUS and C-CEUS were compared using the *χ²* test or Fisher’s exact probability method. TIC parameters were expressed as M (QR), and the non-parametric Mann-Whitney test was used to compare the differences in the two imaging modalities between the two groups. *P* < 0.05 indicated that the differences were statistically significant.

## Results

### General and routine ultrasound characteristics

**Fig. 1:**
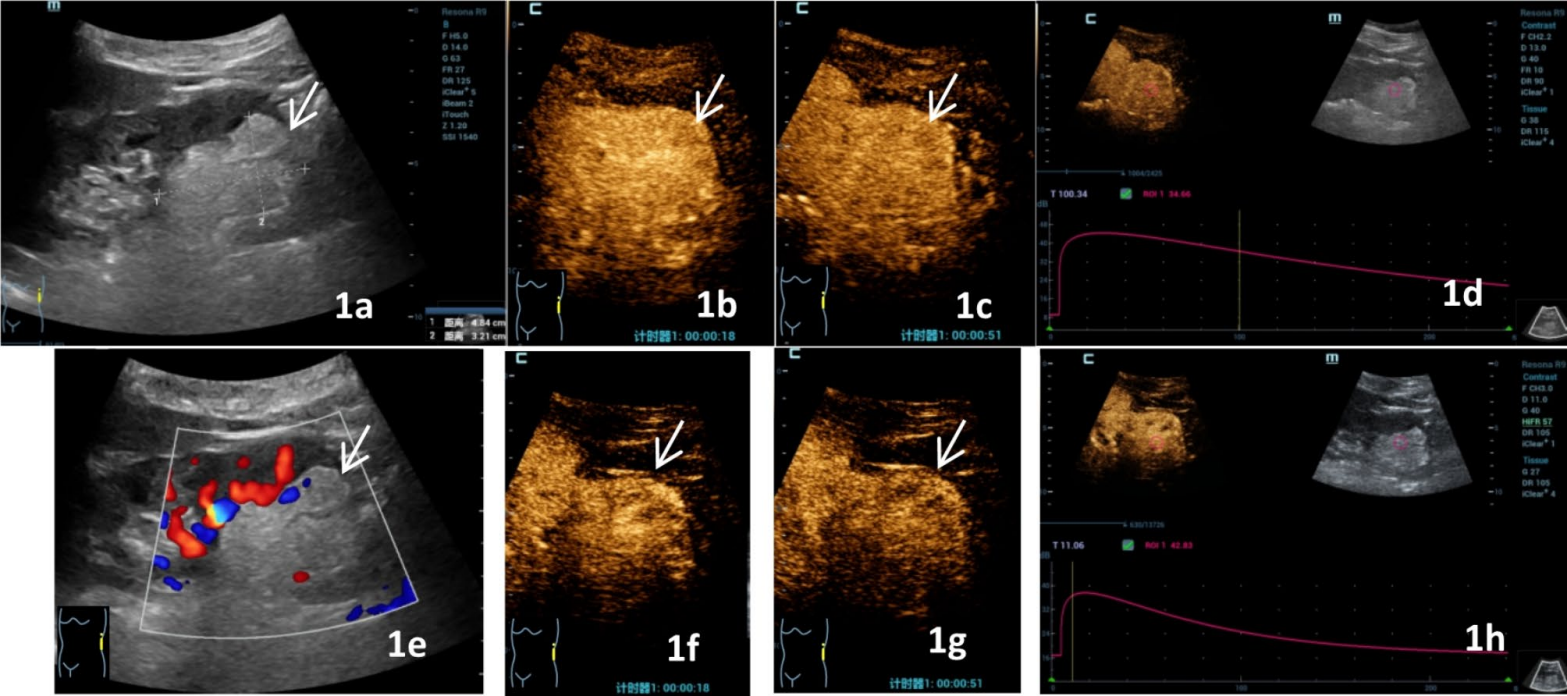
A 35-year-old female patient with angiomyolipoma, gray-scale ultrasound, conventional contrast-enhanced ultrasound (C-CEUS), and high-frame-rate contrast-enhanced ultrasound (H-CEUS) images. **1a**: A hyperechoic mass in the left lower kidney with a size of 4.84 cm × 3.21 cm, with a clear boundary and regular shape (arrow shows the focus). **1b** and **d** show C-CEUS images. **1b**: Homogeneously high enhancement at 18 s during perfusion period. **1c**: homogenous high enhancement at 51 s at wash-out period. **1d**: C-CEUS time-intensity curve. **1e**: CDFI shows grade I blood flow signal in the lesion. **1f** and **h** show H-CEUS images. **1f**: Hermogeneous low enhancement at 18s during perfusion period, **1g**: Hermogeneous low enhancement at 51s during wash-out period. **1h**: H-CEUS time-intensity curve (arrow shows lesions)

A total of 89 masses were included in our study. A total of 30 masses were assigned to the benign group, comprising 18 masses of angiomyolipoma, 2 masses of epithelioid angiomyolipoma, 4 masses of congenital variant, 3 masses of eosinophilic cell tumor, 2 masses of inflammatory nodule, and 1 mass of posterior renal adenoma. The malignant group comprised a total of 59 masses, including 36 masses of clear-cell renal cell carcinoma, 9 masses of papillary renal cell carcinoma, 8 masses of chromophobe cell carcinoma, and 6 masses of other types of renal cell carcinoma. The age and maximum diameter of the nodules of patients in the benign and malignant groups were (57.86 ± 11.87) years vs. (56.53 ± 8.32) years and (4.34 ± 2.19) cm vs. (4.34 ± 2.23) cm, respectively. The two groups’ differences did not reach statistical significance (*t* = 0.549, -0.016, *P* = 0.584, 0.988). Differences in patient gender, surgical approach, and nodal echogenicity,, and CDFI were statistically significant between the benign and malignant groups (*p* = 0.003, < 0.001, < 0.001, 0003, respectively). Male patients were more common in the malignant group, while female patients were more common in the benign group. The malignant group was more likely to show hypoechoic lesions and had more abundant blood flow (55.9% in grade II-III). In the benign group, the lesions were more likely to be hyperechoic with no or little blood flow (73.3% in grades 0-I) (Fig. 1), as displayed in Table 1.

**Table 1:** Characteristics of clinical parameters and conventional US in the malignant and benign groups

		Malignant group(*n* = 59)	Benign group(*n* = 30)	χ²or t	*P* Value
Gender, n (%)	Male	36(61.0%)	8(26.7%)	9.388	0.003
	Female	23(39.0%)	22(73.3%)		
Age (years): mean ± STD		57.86 ± 11.87	56.53 ± 8.32	0.549	0.584
Laterality, n (%)	Left	33(55.9%)	11(36.7.0%)	2.953	0.117
	Right	26(44.1%)	19(63.3%)		
Location, n (%)	Superior	21(35.6%)	8(26.7%)	3.799	0.150
	Middle	19(32.2%)	6(20.0%)		
	Inferior	19(32.2%)	16(53.3%)		
Surgery, n (%)	Radical nephrectomy	23(39.0%)	0(0.0%)	**-**	< 0.001
	Partial nephrectomy	36(61.0%)	19(63.3%)		
	Unoperated	0(0.0%)	11(36.7%)		
Echogenicity, (n/%)	Hyper-	17(28.8%)	25(83.3%)	**-**	< 0.001
	Iso-	2(3.4%)	0(0.0%)		
	Hypo-	40(67.8%)	5(16.7%)		
Boundary, n (%)	Well defined	58(98.3%)	29(96.7%)	-	1.000
	Poorly defined	1(1.7%)	1(3.3%)		
Shape, n (%)	Regular	57(90.9%)	29(90.2%)	**-**	1.000
	Irregular	2(9.1%)	1(9.8%)		
CDFI, n (%)	0	5(8.5%)	9(30.0%)	9.114	0.003
	I	21(35.6%)	13(43.3%)		
	II	13(22.0%)	4(13.3%)		
	III	20(33.9%)	4(13.3%)		
Calcification, n (%)	Yes	4(6.8%)	1(3.3%)	-	0.659
	No	55(93.2%)	29(96.7%)		
Tumor diameter (cm): mean ± STD		4.34 ± 2.19	4.34 ± 2.23	-0.016	0.988

-indicates that the Fisher test was used, with no corresponding statistics

### Comparison of qualitative characteristics between C-CEUS and H-CEUS in the benign and malignant groups

**Fig. 2:**
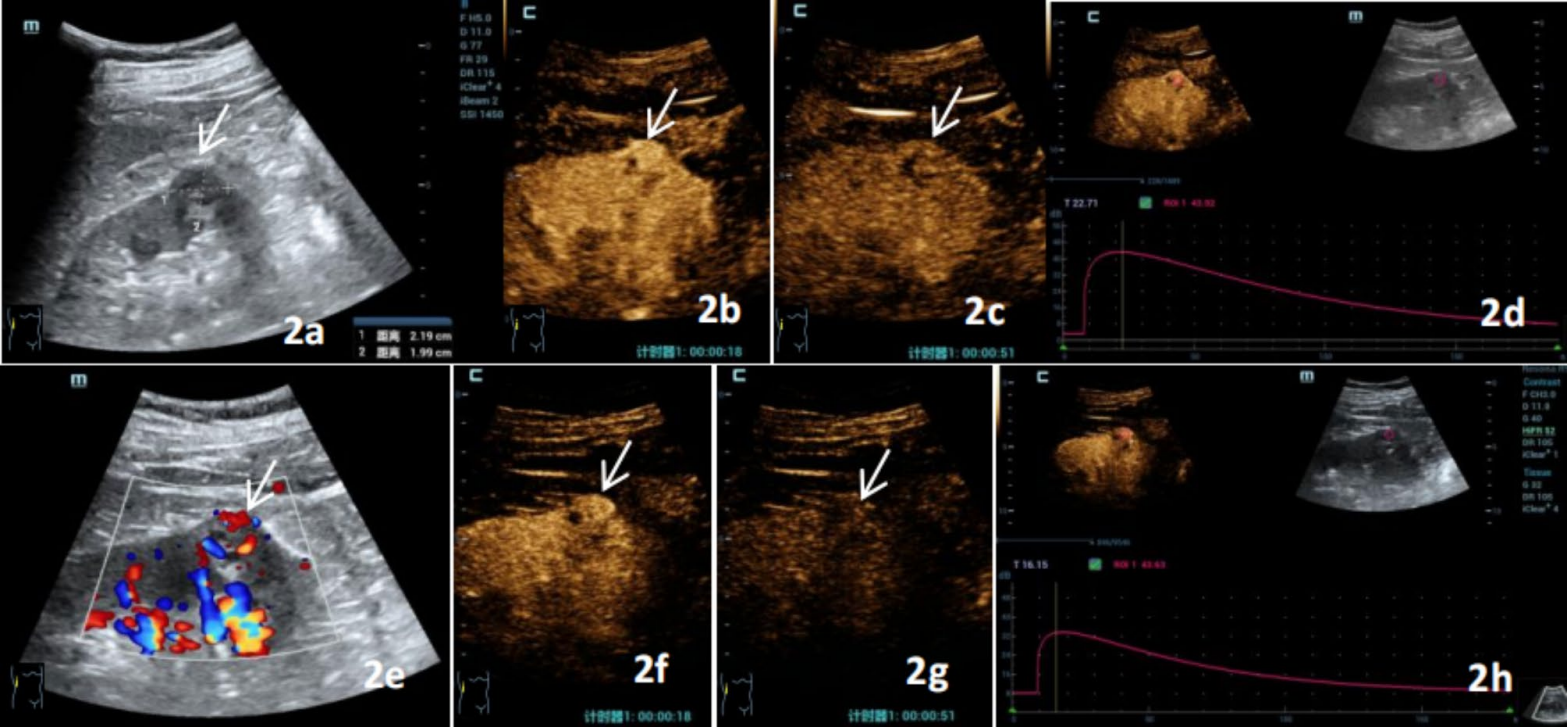
A 45-year-old male patient with clear cell renal cell carcinoma, gray-scale ultrasound, C-CEUS, and H-CEUS images. **2a**: Conventional ultrasound shows extremely hypoechoic mass under the right kidney, with a size of 2.19 cm × 1.99 cm, a clear boundary, and a regular shape. **2b** and **d** show C-CEUS images. **2b**: Diffuse enhancement of both periphery and center of the lesion at 18s during perfusion period. **2c**: Hermogeneous high enhancement at 51 s in the wash-out period. It shows 'fast advance and slow retreat’. **2d**: C-CEUS time-intensity curve. **2e**: CDFI shows grade III blood flow signal in the lesion. **2f** and **h** show H-CEUS images. **2f**: Hermogeneous high enhancement at 18s during perfusion period. **2g**: Hermogeneous low enhancement at 51s during wash-out period.It shows ‘fast advance and fast retreat’. **2h**: H-CEUS time-intensity curve (arrow shows lesions)

Significant differences in the mode of wash-out and fill-in direction were observed in the malignant group between C-CEUS and H-CEUS (*p =* 0.041, 0.002, respectively.) Most of the C-CEUS examinations exhibited a slow wash-out (30/59, 50.8%) and entirety enhancement (32/59, 54.2%), whereas most of the H-CEUS examinations demonstrated fast wash-out (32/59, 54.2%) and centripetal enhancement (43/59, 72.9%) (Fig 2, Table 2). The difference in the homogeneity of enhancement in the benign group was statistically significant (*p* = 0.009), with most of the C-CEUS showing homogeneous enhancement (21/30, 70.0%), whereas most of the H-CEUS examinations revealed hermogeneous enhancement (20/30, 66.7%) (Fig 1, Table 2). However, no significant difference was observed in the mode of wash-out and the direction of enhancement between the two contrast modes in the benign group, which both showed mainly slow wash-out (25/59, 54.2%) and centripetal enhancement (43/59, 72.9%). However, no statistically significant difference in the pattern of wash-out and fill-in direction was found between the two contrast modalities in the benign group, which mainly consisted of slow wash-out (25/30 vs. 30/30) and centripetal enhancement (15/30 vs. 22/30). Furthermore, the two reading sonographers showed strong agreement in the evaluation of the enhancement characteristics of H-CEUS and C-CEUS (Kappa = 0.918 and 0.965 for the benign group and Kappa = 0.934 and 0.925 for the malignant group).

**Table 2:** Qualitative analysis of the features of C-CEUS and H-CEUS in the malignant and benign groups

	Malignant group(*n* = 59)				Benign group (*n* = 30)			
	C- CEUS	H- CUES	*χ²*	*p* Value	C-CEUS	H-CUES	*χ²*	*p* Value
Wash-in mode								
Fast wash-in	45(76.3%)	42(71.2%)	2.796	0.247	1(3.3%)	0(0.0%)	-	0.506
Iso-wash-in	8(13.6%)	5(8.5%)			6(20.0%)	4(13.3%)		
Slow wash-in	6(10.2%)	12(20.3%)			23(76.7%)	26(86.7%)		
Wash-out mode								
Fast wash-out	24(40.7%)	32(54.2%)	-	0.041	5(16.7%)	0(0.0%)	-	0.052
Iso-wash-out	5(8.5%)	0(0.0%)			0(0.0%)	0(0.0%)		
Slow wash-out	30(50.8%)	27(45.8%)			25(83.3%)	30(100.0%)		
Peak intensity								
High enhancement	51(86.4%)	44(74.6%)	-	0.235	9(30.0%)	4(13.3%)	-	0.087
Iso-enhancement	3(5.1%)	4(6.8%)			2(6.7%)	0(0.0%)		
Low enhancement	5(8.5%)	11(18.6%)			19(63.3)	26(86.7%)		
Homogeneity								
Homogeneous	13(22.0%)	12(20.3%)	0.051	1.000	21(70.0%)	10(33.3%)	8.076	0.009
Heterogeneous	46(78.05)	47(79.7%)			9(30.05)	20(66.7%)		
No enhancement								
Yes	45(76.3%)	47(79.7%)	0.197	0.825	3(10.0%)	3(10.0%)	-	1.000
No	14(23.7%)	12(20.3%)			27(90.0%)	27(90.0%)		
Fill-in direction								
Centripetal	27(45.8%)	44(74.6%)	-	0.002	15(50.0%)	22(73.3%)	-	0.110
Entirety	32(54.2%)	15(25.4%)			15(50.0%)	8(26.7%)		
Centrifugal	0(0.0%)	0(0.0%)			0(0.0%)	0(0.0%)		
Boundary after enhancement								
Clear	58(98.3%)	58(98.3%)	-	1.000	30(100.0%)	30(100.0%)	-	1.000
Unclear	1(1.7%)	1(1.7%)			0(0.0%)	0(0.0%)		
Enhancement rang								
Enlarged	1(1.7%)	1(1.7%)	-	1.000	0(0.0%)	0(0.0%)	-	1.000
Unenlarged	58(98.3%)	58(98.3%)			30(100.0%)	30(100.0%)		
Pseudocapsule								
Yes	53(89.8%)	50(84.7%)	0.687	0.582	3(10.0%)	0(0.0%)	-	0.237
No	6(10.2%)	9(15.3%)			27(90.0%)	30(100.0%)		

-indicates that the Fisher test was used, with no corresponding statistics

### Comparison of quantitative characteristics between C-CEUS and H-CEUS in the benign and malignant groups

In the malignant group, the H-CEUS TIC parameters PI, DT/2, AUC, and MTT were lower than in the C-CEUS examination (all *p* < 0.001, Table 3). In contrast, AS was higher in H-CEUS than in C-CEUS (*p* = 0.048, Table 3). In the benign group, the parameters PI, DT/2, AUC, and MTT were lower compared to the C-CEUS examination. All the differences were statistically significant (all *p* < 0.001, Table 3). However, in both the benign and malignant groups, the differences in the H-CEUS TIC parameters AT, TTP, and DS were not statistically significant when compared with C-CEUS (all *p* > 0.05, Table 3); moreover, the differences in the H-CEUS TIC parameter AS in the benign group showed no statistically significant difference when compared with C-CEUS (*p* > 0.05, Table 3).

**Table 3:** Quantitative analysis of the features of C-CEUS and H-CEUS in the malignant and benign groups

	Malignant group(*n* = 59)				Benign group(*n* = 30)			
	C-CEUS	H-CUES	*Z*	*p* Value	C-CEUS	H-CUES	*Z*	*p* Value
AT	9.00(7.20,10.90)	9.17(7.00,10.88)	-0.178	0.859	7.10(5.07,10.20)	6.15(4.45,9.32)	-1.146	0.252
TTP	32.40(25.80,37.60)	28.33(23.91,34.24)	-1.833	0.067	26.60(21.37,36.05)	23.50(17.39,31.86)	-1.094	0.274
PI	47.00(40.57,52.07)	39.67(32.21,46.92)	-3.943	< 0.001	41.66(35.70,48.37)	32.50(28.67,39.99)	-3.548	< 0.001
AS	0.93(0.71,1.09)	1.01(0.77,1.45)	-1.976	0.048	0.79(0.71,0.90)	0.73(0.59,1.09)	-0.274	0.784
DT/2	155.60(131.70, 175.20)	117.37(87.67, 136.33)	-5.315	< 0.001	152.75(123.50, 165.42)	98.87(70.92, 134.36)	-3.667	< 0.001
DS	-0.17(-0.20,-0.14)	-0.17(-0.23,-0.13)	-0.501	0.616	-0.15(-0.17,-0.12)	-0.15(-0.19,-0.11)	-0.059	0.953
AUC	5951.84(4661.48, 7908.28)	2969.96(2360.83, 4062.06)	-6.892	< 0.001	4581.83(3316.67, 5952.74)	1657.03(1214.19,2761.43)	-5.041	< 0.001
MTT	149.10(122.20, 164.80)	99.50(78.61, 129.49)	-5.347	< 0.001	142.90(117.75, 157.95)	90.56(65.30, 124,81)	-3.792	< 0.001

## Discussion

Preoperative diagnosis of benign and malignant renal masses can guide the selection of surgical methods and prevent unnecessary nephrectomy. US be used to distinguish between benign and malignant renal masses by observing their echogenicity, shape, boundaries, capsule, blood flow, etc. Malignant masses typically exhibit hypoechoic lesion, irregular morphology, and abundant blood flow, whereas benign masses typically exhibit hyperechogenicity, regular morphology, and limited blood flow. Similar findings have been observed in this study as well as previously published studies [14], indicating that US can to some extent differentiate the benign and malignant nature of masses. However, not all masses have typical manifestations. For example, AML is a common benign mass with a lack of fat and manifests as a hypoechoic lesion on ultrasound examination. Moreover, malignant masses that are 30-60% small also exhibit hyperechogenicity [15]. In addition, malignant tumors with slow blood flow cannot display rich blood flow signals on conventional ultrasound.

CEUS is a pure blood pool contrast. The contrast agent is made up of phospholipid shell-stabilized gas microbubbles. These 3–7 micron microbubbles are small enough to squeeze past pulmonary capillaries and enter the arterial system but big enough to stay inside blood vessels. The distinction between benign and malignant renal tumors can be made more clearly with CEUS [16, 17] and enables the visualization of vascular perfusion inside the mass. In a meta-analysis of CEUS features in clear cell renal cell carcinoma with a diameter of less than 4 centimeters, Liu et al. [18] suggested that high enhancement has moderate sensitivity (67–89%) and specificity (42–75%), while rapid enhancement and uneven enhancement have high diagnostic ability (AUC of 0.74–0.84). However, unlike other organs in the body, the kidney has a small volume and a large blood flow, reaching up to 20 − 25% of the blood volume of the heart, indicating a high perfusion state. Research [19] suggests that the CEUS examination of renal cell carcinoma mostly demonstrates fast forward and fast backward under high enhancement; however, the majority of kidney tumors exhibit robust blood supply and lack excellent diagnostic specificity. Moreover, the quick perfusion of renal lesions during C-CEUS examination limits the capture of arterial blood flow information, hindering accurate disease diagnosis. By raising the acquisition frame rate, high frame rate contrast-enhanced ultrasonography (H-CEUS) enhanced temporal resolution of images and offered a superior evaluation of vascular enhancement, particularly microvascular enhancement, with upgraded temporal and spatial correlation resolution [20]. Our previous research confirmed that H-CEUS has high sensitivity (84.8%) and specificity (96.8%) in distinguishing between CCRCC and AML [21].

In the benign and malignant groups, two distinct contrast modalities of lesions were compared in this study. The results revealed statistically significant differences in the fill-in direction and wash-out mode of the two ultrasound modes in the malignant group (*P* = 0.041, 0.002). C-CEUS is mainly characterized by entirety enhancement, while H-CEUS is mainly characterized by centripetal enhancement. The difference in enhancement direction between the two contrast modes may be attributed to the increased frame rate, providing a clear image of the specific area of contrast enhancement that first appears inside the lesion. The expansion process of this enhancement area over time directly reflects the perfusion direction of the contrast agent. However, the specific location where the contrast-enhanced area first appears within the lesion cannot be accurately captured in cases with a low image frame rate. Rather, the contrast-enhanced region arises in different areas of the lesion almost simultaneously, resulting in a holistic perfusion effect. In addition, the presence of a large number of immature blood vessels and the higher rate of arteriovenous fistulas in malignant tumors provide oxygen and nutrients to the tumor, promoting tumor growth [22]. Therefore, both C-CEUS and H-CEUS exhibit rapid enhancement. However, the higher time resolution of H-CEUS can improve the frame rate of the image, display the blood flow characteristics of microvasculature more clearly, and improve the differentiating ability of the tumor [23–25]. Therefore, in this study, compared to C-CEUS, H-CEUS was found to more clearly display the initial peripheral enhancement of blood vessels and enhancement gradually move towards the center. Malignant tumors are nodules with abundant arterial blood flow perfusion, showing significantly faster perfusion compared to benign tumors. In addition, H-CEUS can detect the rapid movement of blood flow in arteries within the full field of view. Nonetheless, the benign nodule group did not exhibit a statistically significant variation in the enhancement direction between the two contrast modes. This could be as the benign nodule has fewer blood vessels and a sluggish blood flow perfusion, resulting in both C-CEUS and H-CEUS being able to display its perfusion process.

In malignant tumors, H-CEUS shows rapid wash-out, which is different from the slow wash-out of C-CEUS. The difference in wash-out might explained by the malignant tumors’ abundant blood supply, which requires a longer wash-out time. However, owing to the low frame rate of C-CEUS, the observed initial wash-out time does not reflect the true wash-out time point of the contrast agent inside the tumor. However, H-CEUS can more accurately observe the initial wash-out time of the contrast agent inside the tumor. In benign tumors, due to the limited number of blood vessels, fewer contrast agent microbubbles enter, resulting in poor contrast with the surrounding heterogeneous tissues. However, H-CEUS can display microvessels more clearly, providing a clear contrast with the surrounding necrotic tissue. In addition, In contrast to the surrounding normal renal cortex, which showed equal wash-out in five cases of C-CEUS, the malignant group’s H-CEUS wash-out pattern could be differentiated from it. This could be caused by the substantial improvement in temporal resolution and increase in image information brought about by the switch from 10 Hz in C-CEUS to 50–65 Hz in H-CEUS image capture and frame rate. Especially, H-CEUS reduces contrast agent damage, thereby extending its duration in the late stage and facilitating the observation of the fading mode [26].

The results of this study revealed that in the benign group, only the enhancement homogeneity showed a statistically significant difference between the two contrast modalities (*p* = 0.004), with the benign nodules in C-CEUS showing mainly homogeneous enhancement (21/30, 70.00%), and heterogeneous enhancement (20/30, 66.67%) in H-CEUS mode. This finding may be attributed to benign renal tumors tending to have fewer microvessels and slow flow within them, which are less likely to merge with liquefaction and show homogeneous echoes and homogeneous perfusion of the C-CEUS contrast medium. Nonetheless, by raising the frame rate, the first appearance of contrast enhancement within the lesion can be clearly seen, as well as the direction of contrast agent perfusion and blood flow perfusion; which changes over time and reduces the error of contrast-enhanced ultrasound imaging [27], resulting in the hermogeneous enhancement of the benign group in the H-CEUS mode.

In the TIC parameter analysis of this study, the PI of C-CEUS was higher than that of H-CEUS in both the malignant and benign groups. Moreover, a lower number of contrast microbubbles was accommodated by H-CEUS in the same sampling frame, with a shorter residence time, leading to a lower PI value than that of C-CEUS. Similarly, the higher temporal resolution of H-CEUS enabled accurate observation of the time of entry of contrast microbubbles into the mass and the time of exit. In addition, this duration can be narrowed down, resulting in lower MTT and DT/2 in H-CEUS compared to C-CEUS in both the benign group and the malignant group. Furthermore, the AUC was significantly lower in H-CEUS compared to C-CEUS. In addition, the H-CEUS TIC parameter AS was higher than C-CEUS in the malignant group, and the elevated AS manifested as a steeper and straighter ascending branch of the TIC curve. These results may be attributed to the difference between H-CEUS and C-CEUS; the former had a high frame rate and strong temporal resolution, providing detailed information on the perfusion period of the renal contrast [21]. Malignant renal tumors tend to be blood-rich supply foci, resulting in rapid entry of the contrast agent. In contrast, C-CEUS has a low frame rate and cannot accurately display the contrast agent perfusion process. However, the H-CEUS mode improves the number of image acquisitions from 10 Hz in C-CEUS to 50–65 Hz in H-CEUS, which greatly improves the temporal resolution, enhances image information, and more accurately responds to the process of TIC curve rise.

Nonetheless, the shortcomings of the present study should be acknowledged. (1) The sample size of patients was limited. This study solely examined benign and malignant renal tumors, without grouping renal cancers with different pathological classifications. (2) The study concluded a correlation between tumor size and contrast perfusion process. However, tumor size was not grouped in this study. In the future, the sample will be further expanded and grouped for renal tumors of different classifications and sizes to further clarify the advantages of the application of HFR CEUS. (3) Prospective studies are required to further evaluate the clinical value of H-CEUS technology in the differential diagnosis of the nature of renal tumors.

## Conclusion

CEUS provides a clearer view of the internal enhancement of renal masses and the associated changes in the wash-out period. The quantitative TIC parameters PI, DT/2, AUC, and MTT were lower in H-CEUS compared to C-CEUS. In addition, the qualitative and quantitative analyses revealed that H-CEUS was superior in evaluating the characteristics of solid renal masses compared with C-CEUS.

## Data Availability

Data is available upon reasonable request.
